# HOW FILIAL PIETY INFLUENCES THE LINKS BETWEEN ELDER ABUSE, SOCIAL WELL-BEING, AND DEPRESSION AMONG CHINESE SENIORS

**DOI:** 10.1093/geroni/igad104.2613

**Published:** 2023-12-21

## Abstract

**Objectives:**

Based on Interactive Biopsychological Model, this study examined how multi-dimensional social well-being and filial piety influence the association between elder abuse and depression among older adults in China.

**Methods:**

The participants were 7,700 older adults (aged 60 years or older) enrolled in the 2018 China Longitudinal Aging Social Survey, a national population-based study of older adults. Moderated mediation models were applied to test the mediating effects of multidimensional social well-being (social isolation, loneliness, social network, and social participation) and the moderating effect of filial piety. All mediation and moderated mediation effects were estimated using SPSS.26. Results: Different dimensions of social well-being have a partial mediating effect on the association between elder abuse and depression. Traditional attitudes towards filial piety exacerbated the effects of elder abuse on depression by improving social isolation and loneliness while reversing the negative effects of elder abuse on depression through improving the social network. The interaction of elder abuse and filial piety has no significant influence on social participation. Conclusions: Although particular sociodemographic factors are associated with a greater risk of depression for elder abuse victims, our findings suggested that victims with traditional attitudes towards filial piety are more likely to feel isolated subjectively (lonely, worse social adaptation) and more depressed. This should be taken into consideration when developing culturally sensitive identification, prevention, and intervention services for older adults.

